# Arthroscopical and histological study of cartilaginous lesions treated by mosaicplasty

**Published:** 2010-11-25

**Authors:** RD Rădulescu, CF Cirstoiu, AE Bădilă

**Affiliations:** Orthopedics and Traumatology, Bucharest University Hospital, BucharestRomania

**Keywords:** arthroscopy, minibiopsy, histopathology, osteocartilaginous autografts

## Abstract

**Aim.** The aim of our study was to assess macro– and microscopically the knee cartilaginous lesions outcome treated by mosaicplasty.

**Material and method** Our study included 32 patients which underwent mosaicplasty for nondegenerative cartilaginous lesions of the knee and a second look arthroscopy. In 21 patients, minibiopsies from the repaired lesion were performed under arthroscopic control (from the cartilaginous region of the transplanted osteocartilaginous grafts and from the spaces between grafts). All repaired lesions were carefully examined during arthroscopy and all harvested minifragments were studied by optical microscopy (staining method –  hematoxylin eosin).

**Results** Macroscopically, the articular surface of the repaired cartilaginous lesions was smooth and congruent to the adjacent surfaces. The aspect and resistance to compression of grafted area was similar to those of the normal surrounding cartilage. The transferred cartilage maintained its height, being at the level of the neighboring cartilage. One year postoperatively, the limits of the cartilaginous autografts were still visible. Two years postoperatively, these limits were no longer visible. Microscopically, the region of the former lesion was constituted mainly by viable hyaline cartilage. Fibrous cartilaginous tissue was visualized in the spaces between the grafts.

**Conclusions** The second look arthroscopy showed that after mosaicplasty the repaired articular surface was smooth, leveled, homogenous and congruent to adjacent cartilage. The spaces between grafts are progressively covered by fibrous cartilaginous tissue with a more textured and uneven surface. Mosaicplasty is a biological surgical technique which restores the normal osteocartilaginous architecture of the most part of the grafted area. The transplanted osteocartilaginous cylindrical grafts maintain its viability and mechanical properties.

## Introduction

The hyaline cartilage is very resistant in time to load and usage, but once injured, its regenerating capacity is limited. The problem is still more acute in young patients, whose active life style subjects their joints, especially those of the legs, to great stress. Modern society led to the disappearance of traditional life style where hard work was predominant, but on the other side accelerated the pace of life, prolonged the period of activity, both during a day and throughout life. The workday often surpasses the light day and the practically unlimited availability of hyper–calorigen foods led to ever increasing overweightness prevalence among all age groups; modern medicine has led to a substantial increase in average life span and thus the joints ‘working’ time increased accordingly. The number of joints loading cycles doubled or even tripled, and the human species could not adapt to these changes of demands to which the body is subject to, changes that took place in a very short time on the evolution scale. All these elements show the importance given to the joint pathology in all its aspects: etiology, prevention, diagnosis, conservative and surgical treatment, possible complications, prognosis, as well as to the related areas: physiopathology, pathogenesis, joint biomechanics, etc.

One of the modern treatment methods of cartilaginous and osteocartilaginous injuries of the bearing surfaces (osteocartilaginous fractures, dissecting osteocondritis – Konig disease, posttraumatic cartilage injury, etc.) is to transfer cylinder shaped, small, but numerous osteocondral autografts, from the non–bearing zones to the bearing ones (mosaicplasty)[3,4,5]. Thus it is possible to restore the shape of the articular surface ensuring a superior articular congruence both to the adjacent cartilage and to the one vis–a–vis. The advantage of this technique is the integration of the spongious elements of graft, which fuse with the spongious bed of the host surface and ensure a strong attachment of the autografts. Another advantage is the transfer of very high quality hyaline cartilage to the affected area[6].

## Purpose

The aim of the study is to evaluate from a hystological point of view the post–operative results of mosaicplasty on a medium term basis. 

## Material and method

**Figure 1:**
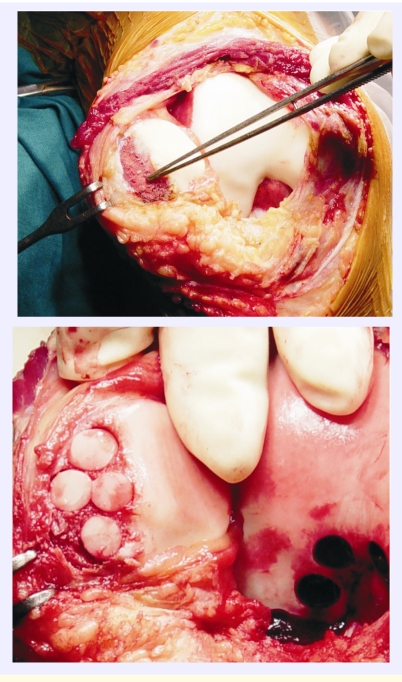
Mosaicplasty for an osteocartilaginous fracture of the patella

Our study is a prospective one, extending on a 10 years period (2000 – 2009). During this period, in the Orthopedics and Traumatology Clinic within the University Emergency Hospital 83 mosaicplasties (Figure 1)  have been carried out for cartilaginous or osteocartilaginous lesions of the knee. Of these, 78 patients were available for assessment of final results. In 32 cases a second look arthroscopy was performed.

In 21 of them mini–biopsies have been practiced from the rebuilt injuries during some control arthroscopies. The macroscopic examination was performed arthroscopicaly and the collected fragments were stained with hematoxylin eosin and analyzed in optical microscopy.

The sex distribution was 13 males / 8 females. The average age was 28 years and 5 months with limits between 18 and 57 years.

Chondral or osteochondral lesion etiology was: traumatic, osteonecrosis (dissecting osteochondritis, Konig disease) or microtrauma. The lesions consisted of posttraumatic chondral lesions–5, osteocartilaginous fractures–6, dissecting osteochondritis–5.

## Results

### The macroscopic aspect on control arthroscopy

**Figure 2:**
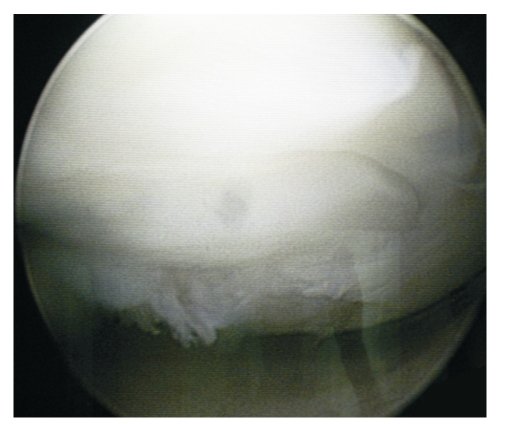
Arthroscopical macroscopic view of an osteochondral plug at 8 months postmosaicplasty

In 32 cases arthroscopies were practiced (second look), evaluating the overall shape and aspect of the area, the quality of the cartilage, the fibrocartilage formation in the space between the osteocartilaginous autografts, the integration of the grafts in the adjacent tissues. The hyaline cartilage of the grafts, the one in the area adjacent to the lesion and the intercalating cartilage were palpated. The indications for second look arthroscopy were: knee injury that raised the suspicion of an intra–articular injury (meniscus, crossed ligaments, a new chondral or osteochondral lesion) and the need for a new surgical intervention (arthroscopic ligamentplasty, arthroscopic shaving, etc.). Arthroscopically, the osteocartilaginous cores preserved the level at which they were implanted, were not blocked and did not become prominent (Figure 2).

The cartilaginous component maintained its smooth appearance of the articular surface, its color, its elastic resistance. There was no loss of cartilage in the transplanted area. In the spaces between the grafts a neocartilaginous tissue is observed, which can be pressed on palpation with the explorer, having heterogeneous consistency. Overall, the area is smooth and congruent.

When arthroscopy was carried out at approximately 1 year postoperatively, the autografts cartilaginous component was still clearly visible, revealing a discrete ditch at the limits between it and the adjacent cartilage. At 2 years postoperatively, this limit was no longer visible.

According to the file of cartilage repair assessment (International Cartilage Repair Society–Cartilage Repair Assessment), 24 patients were classified as grade Ⅰ (normal cartilage) and 8 as grade Ⅱ (almost normal cartilage).

Inspection of donor areas showed progressive their progressive obliteration with fibrocartilage. In 3 cases the fibrillation of the pericanalar cartilage was observed.

### Histopathology of the arthroscopically collected fragments

In 21 cases minimal cartilaginous biopsy was practiced from the osteocartilaginous grafts and from the spaces between the grafts. The hystopathological examination showed the existence of a viable hyaline cartilaginous tissue in the cartilaginous component of the osteochondral autografts and fibrous cartilaginous tissue in the spaces between the grafts. 

11 of the minibiopsies were taken at approximately 1 year postoperatively (on average 12.4 months). At this point, the osteocartilaginous cores were still visible and it was possible to make targeted collection of core or fibrocartilage formed in the intercalary spaces. 2 pieces were taken from the osteocartilaginous autografts and 2 from the fibrocartilage.

**Figure 3:**
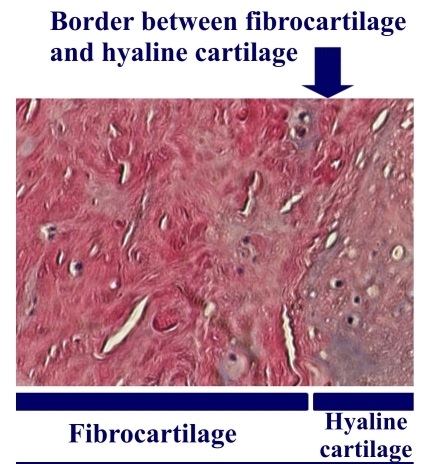
Histological view of the border between hyaline cartilage and fibrocartilage at the limit of an osteochondral graft

10 of the minibiopsies were taken at almost 2 years postoperatively (on average 21.67 months), when the osteochondral autografts were perfectly integrated with the hyaline osteoarticular adjacent cartilage and with the fibrocartilage between them. Thus, there was not the possibility to individualize them, and the collection of fragments was from the area of the reconstructed lesion – 6 pieces (3 central, 3 peripheral). In the 60 minifragments collected the following were found:

- hyaline cartilage in 42 minifragments (70,00%);
- fibrocartilage in 13 (21,67%);
- hyaline cartilage – fibrocartilage association and boundary areas in 5 (8,33%) (Figure 3)

We can consider we have emphasized hyaline cartilage in 42 + 0,5 x 5 = 44,5 fragments–74,17% of the surface and fibrocartilage in 25,83% of the collecting surface.

## Discussion

In cartilaginous and osteocartilaginous injuries, the purpose of the treatment is to obtain a painless, mobile and resistant joint, keeping these features stable over time. This can be achieved by restoring an articular surface congruent with the adjacent cartilage and with a mirrored articular surface, whose mechanical properties make it resistant to existing biomechanical stress in the respective joint[1,2]. Untreated lesions tend to worsen and lead to secondary degenerative lesions both in the injured area and on the surface vis–a–vis, compromising the biomechanical axis and the movements in the respective joint and leading to the extension of arthrosic lesions to the entire joint. In order to prevent degenerative lesions, the post–traumatic or post–necrotic cartilaginous lesions must be treated early in their development and thoroughly [7,8,9,10]. 

The control arthroscopy revealed the restoration of cartilage surface, which is continuous, smooth, rather homogenous, resistant and congruent with the adjacent or counterlateral articular surfaces. The aspect and the color of the cartilage are quasinormal. Palpation indicates stronger areas, corresponding to the grafted osteocartilaginous cores and weaker areas–corresponding to the fibrocartilage which came to cover the intercalary spaces. If one year after surgery, the osteocartilaginous autografts are easily identifiable, at 2 years postoperatively their boundaries are blurred. No elevations or clogging of the autografts was observed.

The hyaline articular cartilage has a multistratified structure:

- lamina splendens is the most superficial layer–it is acelular;
- the tangential layer–chondrocytes are small and flat parallel to the surface. Collagen fibers are very fine and are also parallel to the surface.
- transitional area–chondrocytes are big, round, isolated or grouped. The collagen fibers are oriented obliquely
- radial area–chondrocytes are larger than the ones in the previous layers and are grouped in columns perpendicular to the articular surface. The collagen fibers are parallel to the chondrocytes columns.
- calcified cartilage layer. It is the deepest layer, and is in direct contact with the subchondral bone.

The hystopathological examinations carried out on the minifragments collected have proven the existence of hyaline tissue having exactly this structure.

The hyaline cartilage is a connective tissue formed of its classical components: cells and extracellular matrix made of the fundamental substance and connective fibers.

Under the standard conditions of optical microscopy the matrix appears unstructured because the fibers are under the resolution limit of the optical microscopy (aprox 20nm), and the refraction index is approximately equal to that of the fundamental substance. 

**Figure 4:**
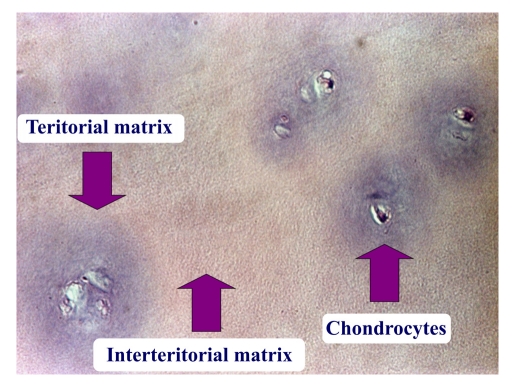
Hyaline cartilage postmosaicplasty at the level of the lesion at 7 months postsurgery–hematoxyline–eosine 200X

**Figure 5:**
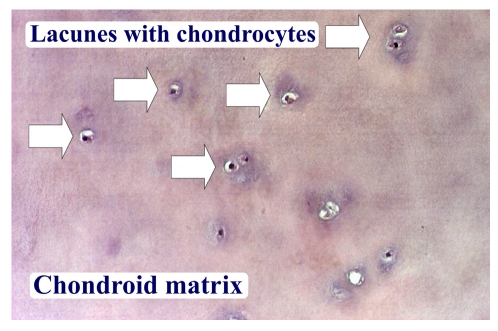
Hyaline cartilage postmosaicplasty at the level of the lesion at 1 year after surgery–hematoxyline–eosine 200X

In the articular hyaline cartilage the chondrocytes are gathered in so called ‘isogenic groups’. The matrix around these isogenic groups is called territorial matrix. It contains larger quantities of glucoseaminoglycans than the rest of the matrix, and under hematoxylin eosin coloration appears more basophilic and is more intensely, darker colored. The rest of the matrix is called extraterritorial matrix (Figure 4). The chondrocytes occupy gaps in the fundamental substance–the superficial gaps contain one chondrocyte each, the ones found deeper can contain more (Figure 5).

Under Van Gieson coloration, the collagen is colored in red. The territorial matrix appears lighter in color, and the extraterritorial one more intense, darker. Hematoxylin and eosin color the territorial matrix darker; thus, the isogenic groups of chondrocytes have a darker halo around them. 

The fibrocartilage contains less chondrocytes, these are flat, isolated or in slam isogenic groups. It is richer in collagen fibers. 

In order not to damage the restored surface, the minibiopsies were carried out with a very fine puncture needle. The hystopathologic examination of the chondral minibiopsies showed that the surface is 75% covered in average by hyaline cartilage and 25% in fibrocartilage. The viability and high quality of the transplanted hyaline cartilage and its very good integration with the neighboring fibrocartilage was observed. 

## Conclusions

1. Mosaicplasty is the only surgical technique which ensures the transfer of autologous hyaline cartilage, structured and viable, at the lesion level, allowing the restoration of articular congruency.
2. On the control arthroscopic examination, the restored articular surface is smooth and homogenous. The hyaline cartilage of the autografts is viable, smooth, firm, homogenous, of normal color. The intercalary spaces are progressively covered by cartilaginous tissue having an non–homogenous aspect and a less regular surface.
3. One year postoperatively, the cartilaginous component of the autografts is still clearly visible, showing a discrete ditch at the limit between it and the adjacent cartilage. Two years postoperatively, this limit is no longer visible.
4. Hystopathologicaly, the surface of the former lesion consists of 74% hyaline cartilage and 26% fibrocartilage

## References

[1] Bădilă A, Rădulescu R, Niţă C, Nutiu O (2008). Microfractures versus mosaicplasty in the treatment of nondegenerative chondral and osteochondral of the knee. Revista de Ortopedie si Traumatologie.

[2] Bădilă A, Rădulescu R, Niţă C, Nuţiu O, Manolescu R (2008). Resistence of fixation of cilindrical osteocartilaginous grafts in mosaicplasty technique. Revista de Ortopedie şi Traumatologie.

[3] Hangody L, Fules P (2003). Autologous osteochondral mosaicplasty for the treatment of full thickness defects of weight bearing joints–ten years of experimental and clinical experience. JBJS A.

[4] Hangody L (2000). Mosaicplasty. Surgery of the Knee.

[5] Hangody L, Vasarhelyi G, Hangody  LR (2008). Autologous osteochondral grafting–technique and long–term results. Injury.

[6] Marcacci M, Kon E, Delcogliano  M (2007). Arthroscopic autologous osteochondral grafting for cartilage defects of the knee: prospective study results at a minimum 7–year follow–up. Am J Sports Med.

[7] Rădulescu R, Cirstoiu C, Bădilă A, Nuţiu O (2007). Mosaicplasty in the treatment of osteocartilaginous lesions of the knee. Revista Medico–Chirurgicala a Societatii de Medici si Naturalisti din Iasi.

[8] Rădulescu R, Cirstoiu C, Bădilă A (2007). Treatment of isolated cartilaginous lesions of the knee by mosaicplasty (abstract). The Romanian Anti–aging Magazine.

[9] Rădulescu R, Cirstoiu C, Bădilă A, Nuţiu O (2006). Results of mosaicplasty in osteocartilaginous lesions of the knee. Medic.ro.

[10] Rădulescu R, Bădilă A, Cirstoiu C, Nuţiu O (2006). Microfractures versus mosaicplasty in the treatment of osteocartilaginous fractures of the knee. Chirurgia.

